# Prostate cancer screening based on genotyping for high risk founder alleles

**DOI:** 10.1186/1897-4287-10-S4-A28

**Published:** 2012-12-10

**Authors:** Cezary Cybulski, Dominika Wokołorczyk, Wojciech Kluźniak, Jan Lubiński

**Affiliations:** 1 Department of Genetics and Pathomorphology of the Pomeranian Medical University, Szczecin, Poland

## 

Inherited factors contribute to the lifetime risk of prostate cancer. These factors include a positive family history of cancer and a mutation in one of several prostate cancer susceptibility genes, A number of genome-wide association studies (GWAS) have identified over a number of single nucleotide polymorphisms that have been confirmed to be associated with prostate cancer risk We evaluated whether or not genotyping of 18 different prostate cancer founder alleles in the Polish population is helpful in identifying high-risk individuals and for determining optimal screening regimens. A serum PSA level was measured and a digital rectal examination was performed on 2907 unaffected men aged 40-90. All men were genotyped for three founder alleles in BRCA1 (5382insC, 4153delA, C61G), four alleles in CHEK2 (1100delC, IVS2+1G>A, del5395, I157T), one allele in NBS1 (657del5), one allele in HOXB13 (G84E), and for nine SNPs which have previously been shown to be associated with prostate cancer risk. A founder mutation in CHEK2 (I157T) predicted prostate cancer in unscreened men.

